# Predictive Factors of Severe Adverse Events in Pediatric Oncologic Patients with Febrile Neutropenia

**DOI:** 10.31557/APJCP.2020.21.12.3487

**Published:** 2020-12

**Authors:** Jutarat Thangthong, Suvaporn Anugulruengkitt, Supanun Lauhasurayotin, Kanhatai Chiengthong, Hansamon Poparn, Darintr Sosothikul, Piti Techavichit

**Affiliations:** 1 *Department of Pediatrics, Faculty of Medicine, King Chulalongkorn Memorial hospital, Chulalongkorn University, Bangkok, Thailand. *; 2 *Division of Pediatric Infectious Diseases, Department of Pediatrics, Faculty of Medicine, Chulalongkorn University, Bangkok, Thailand. *; 3 *Center of Excellence for Pediatric Infectious Diseases and Vaccines, Chulalongkorn University, Bangkok, Thailand. *; 4 *Division of Pediatric Hematology and Oncology, Department of Pediatrics, Faculty of Medicine, Chulalongkorn University, Bangkok, Thailand. *; 5 *STAR Pediatric Hematology and Oncology, Department of Pediatrics, Faculty of Medicine, Chulalongkorn University, Bangkok, Thailand. *

**Keywords:** Febrile neutropenia, neutropenic fever, pediatric cancer

## Abstract

**Objectives:**

Febrile neutropenia (FN) is severe and potentially life-threatening in oncologic patients. The objective of this study is to define the factors associated with severe adverse outcomes of pediatric FN.

**Methods:**

A retrospective and prospective descriptive study performed in pediatric patients diagnosed with FN at King Chulalongkorn Memorial Hospital from January 2013 to December 2017. Severe adverse events defined as the presence in one of these following oxygen therapies, mechanical ventilator, shock, admission to ICU, renal dysfunction, and liver dysfunction.

**Results:**

The study included 267 patients with 563 febrile neutropenia episodes. The median (range) age was 5.1 years (1 month-15 year). Among 563 febrile neutropenia episodes, 115 episodes (20%) developed severe adverse events. The FN patients were classified into low and high-risk groups, 91% of patients with severe adverse events and all 21 patients who died were in high risk group. The overall mortality rate was 3.1%. Factors associated with severe adverse events were fungal infection (aOR 6.51, 95%CI 2.29-18.56), central venous catheter insertion (aOR 4.28, 95% CI 2.51-7.29), CPG defined high risk (aOR 3.35, 95%CI 1.56-7.17), viral infection (aOR 2.72, 95%CI 1.05-7.06), lower respiratory tract infection (aOR 2.52, 95%CI 1.09-5.82) and treatment not according to CPG (aOR 2.47, 95% CI 1.51-4.03).

**Conclusions:**

Fungal and viral infection, central venous catheter insertion, lower respiratory tract infection, CPG defined high risk and treatment not according to CPG were associated factors of increased risk for severe adverse events. Our current institutional CPG for FN in children was applicable and improved clinical outcomes for this group of patients.

## Introduction

Febrile neutropenia (FN) is a common problem which is seriously and potentially life-threatening in the patient who received chemotherapy (Lehrnbecher et al., 2017). Prompt treatment and administration broad-spectrum intravenous antibiotics are necessary to reduce mortality and improve outcomes in treatment (Das et al., 2016). 

It is crucial to develop the risk-stratification models to identify the patients at low and high risk of severe complications to deliver the most appropriate management to individual patients (Klastersky, 2004; Sipsas et al., 2005). Our institution has developed the clinical practice guideline for FN since 2013, which focuses on FN in children and adolescents with cancer. It should be appropriate to administer less aggressive treatment in low-risk patients to avoid treatment-related complications such as multidrug-resistant and prolong hospitalization (Klastersky, 2004; Lucas et al., 2018). 

Previous studies classified patients into low and high-risk groups to develop adverse events using multiple clinical factors(Lehrnbecher et al., 2017; Freifeld et al., 2011). High-risk group define in patients with neutropenia (ANC <500 cell/m^3^) anticipated to last more than seven days, evidence of hepatic or renal insufficiency, infants with acute lymphoblastic leukemia, patients with acute myeloid leukemia (Hakim et al., 2010), patients within 30 days of hematopoietic cell transplant (HCT) and comorbid medical problems (such as hemodynamic instability, gastrointestinal symptoms, signs of intravascular catheter infection) (Rondinelli et al., 2006; Ammann et al., 2010).

The objective of this study is to define the factors associated with severe adverse outcomes in pediatric patients with FN using our institutional clinical practice guideline.

## Materials and Methods

*Study population and design*

This study was conducted at Department of Pediatrics, King Chulalongkorn Memorial Hospital (KCMH), Bangkok, Thailand. Retrospectively review of the medical records from January 2013 to December 2017 using ICD-10 code: D70 (agranulocytosis) in patients aged less than 15 years were performed. Clinical data collection includes age, gender, underlying disease, stage of cancer, types of cancer, status of disease, chemotherapy regimens, time of fever prior to admission, prophylaxis granulocyte stimulating growth factor (G-CSF), treatment, and complications. 

*Definition*

Febrile neutropenia was defined as a single oral body temperature (BT) greater than 38.3^o^C or more than 38.0^o^C and sustain 1 hour (Freifeld et al., 2011) while neutropenia defines as absolute neutrophil count (ANC) less than 500 cells/mm3 or ANC less than 1,000 cells/mm3 and predict to decrease until less than 500 cells/mm3 within 48 hours later (Freifeld et al., 2011).

Severe adverse events defined as the presence in one of these following oxygen therapy, mechanical ventilator, shock, admission to intensive care, renal dysfunction, and liver dysfunction (Anirban et al., 2016; Prasad et al., 2014). Shock defined as systolic blood pressure lower than the 5th percentile for an age-matched normal range required fluid resuscitation or inotropic agents to raise blood pressure (Balazs et al., 2017). Hypoxia defined as oxygen saturation lower than 90% using a pulse oximeter (Wilson et al., 2010). Renal dysfunction was defined as serum creatinine greater than 0.5 mg/dL or raising above two times of upper limit for age or raising 2-fold increase in baseline creatinine value (David A et al., 2020). Hepatic dysfunction was defined as total bilirubin greater than 4 mg/dL, alanine transaminase level above two times upper limit for age (David A et al., 2020). Mortality rate was assessed within 2 weeks of the onset of febrile neutropenia.

*KCMH clinical practice guideline for management of febrile neutropenia*

According to our institutional clinical practice guideline, the eligible patient is patient who present with acute febrile illnesses which body temperature higher than 38 degree Celsius and had one of the following conditions include chemotherapy or radiotherapy treatment or history of bone marrow transplantation. These patients will be immediately evaluated for vital signs and clinical symptoms of severe infection and will be investigated as septic workup and received intravenous ceftriaxone within one hour. Patients categorize into a high- and low-risk group using risk assessment group criteria. All high-risk patients will receive treatment with piperacillin/tazobactam, while low-risk patients will receive ceftazidime plus amikacin if ANC <500 cells/mm3.

**Figure 1:**
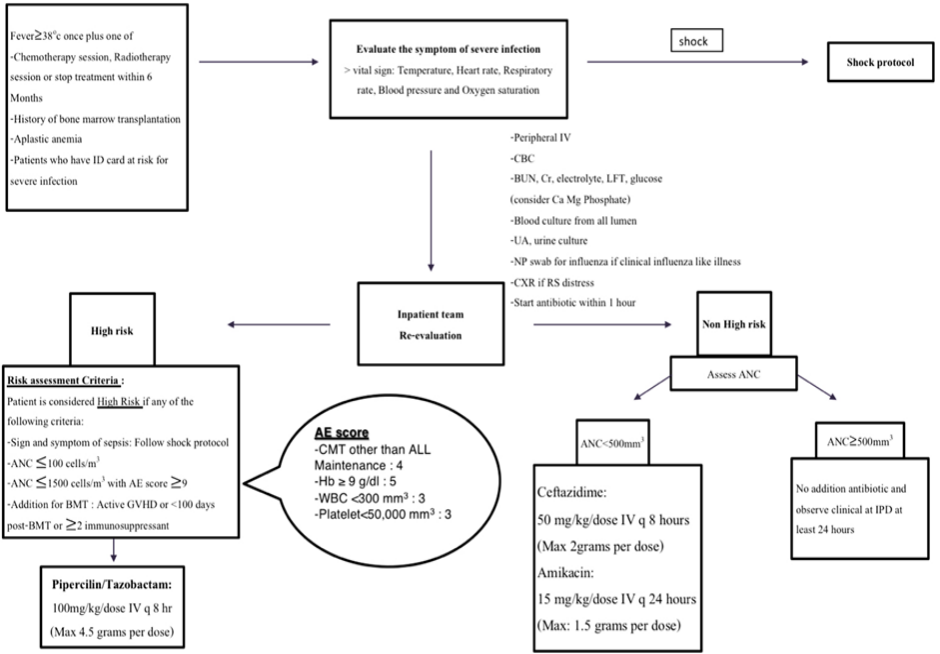
Clinical Practice Guideline for the Management of Febrile Neutropenia at King Chulalongkorn Memorial Hospital

The patient defines as high-risk by one of the following: signs and symptoms of sepsis, ANC less than 100 cells/mm^3^, ANC less than 1,500 cells/mm^3^ with adverse event (AE) score more than 9, underwent BMT within 100 days, active graft versus host disease, receive more than two immunosuppressants. AE score is the summation of the following clinical scores, which are chemotherapy other than maintenance phase of acute lymphoblastic leukemia (4 points), hemoglobin higher than 9 g/dl (5 points), WBC less than 300 cells/mm^3^ (3 points), and platelet less than 50,000 cells/mm^3^ (3 points) (Figure 1). 

Treatment not according to CPG defined as all episodes that not received medication in low-or high- risk group and not follow each steps of managements.

*Statistical analysis*

Categorical variables were expressed as numbers and percentages. Continuous variables were expressed as medians and ranges. Risks factors associated with severe adverse events were compared using Mann-Whitney test or Fisher’s exact test as appropriate. We assessed the potential association of factors associated with severe complications of febrile neutropenia by using univariate and multivariate logistic regression analysis. Statistical analysis was performed using SPSS version 22. The univariate analysis that p-value <0.1 were considered to analyze by multivariate analysis and p-value <0.05 was considered significant.

## Results

*Patient’s characteristics*

From January 2013 to December 2017, there was 1,244 admissions according to ICD-10 code D63.0, D70.1 and D69.6 with a diagnosis of anemia in neoplastic disease, agranulocytosis and thrombocytopenia. Seven hundred ninety-six admissions were excluded because of repeated patients and a lack of confirmed diagnosis of febrile neutropenia. A total of 267 patients with 448 admission and 563 FN episodes were included. The median (range) age of patients were 5.1 years (1 month - 15 years) with male predominate (60%). One hundred ninety-one patients (71.5%) had hematologic malignancy including ALL, AML and lymphoma. Patients demographic data were shown in Table 1.

*Febrile neutropenia episodes*

The characteristics of FN episodes were shown in table 2. Median (range) time of fever prior to admission was 1 (0-60) day. Median (range) time from the most recent chemotherapy treatment was 10 (0-148) days. Median (range) duration of neutropenia was 8 (1-49) days. At the time of FN diagnosis, median (range) of hemoglobin (Hb) was 9.5 (2.7-26) g/dl, white blood cell counts 670 (10-235,150) cells/mm^3^, absolute neutrophil counts 70 (0-9,522) cells/mm^3^ and platelet 79,092 (1,000-601,000) cells/mm^3^. Two hundred seventy-nine episodes (49.6%) had fever without localizing source of infection, 192 (34.1%) had microbiologically documented infections and 92 episodes (16.3%) had clinically documented infection. Among microbiologically documented infection, there were 154 (80%) bacteria, 32 (16%) virus, 27 (14%) fungus and 1 (0.5%) Mycobacterium tuberculosis. Lower respiratory tract infection was found in 13 (2.3%) episodes. 

According to our clinical practice guideline, 426 (76%) episodes were defined as high risk group. Three hundred seventy-three (66%) patients receive treatment follow KCMH clinical practice guideline. 

*Severe adverse events*

**Figure 2:**
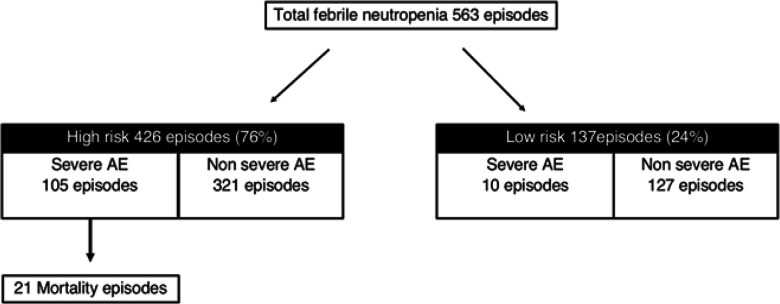
Risk Categorized of Febrile Neutropenia by KCMH Guideline

Among 563 FN episodes, there were 115 (20%) episodes developed severe adverse events which 105 episodes of these were in the high-risk group. Risk categorized of febrile neutropenia by KCMH guideline was shown in Figure 2. Severe adverse events were patients needed oxygen therapy in 110 (95.6%), shock in 100 (86.9%), and admitted in intensive care in 78 (67.8%) episodes. Renal and liver dysfunctions found in 24 (20.8%) and 8 (6.9%) episodes respectively. The summarized of severe adverse events were shown in Table 3. Twenty-one patients were died, which all of these were high risk group.

*Risk factors of severe adverse events*

Risk factors of severe adverse events were shown in Table 4. AML, CPG defined high risk patients, induction chemotherapy, central venous catheter usage were significantly associated with severe adverse outcome (P <0.001 and P < 0.001 respectively). 

The univariable analysis of factors associated with severe adverse events of febrile neutropenia were shown in Table 5. Factors associated with severe adverse events of febrile neutropenia were acute myeloid leukemia (OR 1.86, 95%CI 1.16-2.95), lower respiratory tract infection (OR 5.34, 95% CI 2.58-11.04), fungal infection (OR 7.88, 95% CI 3.16-20.76), central venous catheter insertion (OR 5.53%, 95% CI 3.44-8.99), CPG defined high risk (OR 4.15, 95% CI 2.08-9.19) and treatment not according to CPG (OR 2.07, 95% CI 1.33-3.22). Patients with standard risk of ALL have a significant lower risk of severe adverse events during the treatment (OR 0.44, 95%CI 0.18-0.96).

Furthermore, multivariable analysis found factors associated with severe adverse events (Table 5) were fungal infection (aOR 6.51, 95%CI 2.29-18.56), central venous catheter insertion (aOR 4.28, 95% CI 2.51-7.29), CPG defined high risk (aOR 3.35, 95%CI 1.56-7.17), viral infection (aOR 2.72, 95%CI 1.05-7.06), lower respiratory tract infection (aOR 2.52, 95%CI 1.09-5.82) and treatment not according to CPG (aOR 2.47, 95% CI 1.51-4.03).

**Table 1:** Demographic Data of 267 Patients with Febrile Neutropenia

Patient characteristics	Number (%)
Age, years (median, range)	5.1 (1 month - 15 years)
Male	151 (56.6)
Chemotherapy	
Induction for leukemia	75 (27.5)
Other chemotherapy	192 (72.5)
Underlying diseases	
Hematologic malignancy	191 (71.5)
Acute lymphoblastic leukemia	114 (42.7)
Acute myeloid leukemia	44 (16.5)
Lymphoma	20 (7.5)
Other*	13 (4.8)
Solid tumor	76 (28.5)
Brain tumor	23 (8.6)
Neuroblastoma	17 (6.4)
Rhabdomyosarcoma	7 (2.6)
Osteosarcoma	5 (1.9)
Hepatoblastoma	4 (1.5)
Ewing sarcoma	4 (1.5)
Retinoblastoma	4 (1.5)
Other**	12 (4.5)

* Hemophagocytic lymphohistiocytosis, Langerhans cell histiocytosis, Chronic myeloid leukemia, Juvenile myelomonocytic leukemia; **, Wilms’ tumor, Primitive neuroectodermal tumor, Extrarenal rhabdoid tumor, Small cell lung cancer, Soft tissue sarcoma, Atypical teratoid rhabdoid tumor, Yolk sac tumor, Mix germ cell tumor

**Table 2:** Baseline Characteristics of 563 Febrile Neutropenia Episodes

Febrile neutropenia characteristics	
Median (range) time of fever prior to admission (days)	1 (0,60)
Interval since last chemotherapy (days) (Median, range)	10 (0,148)
Laboratory results at the time of febrile neutropenia diagnosis– Median (range)	
Hemoglobin, g/dl	9.5 (2.7-26)
White blood cell count, cells/mm^3^	670 (10-235,150)
Absolute neutrophil count, cells/mm^3^	70 (0-9,522)
Platelet, cells/mm^3^	79,092 (1,000-601,000)
Duration of fever in days (mean±SD)	4.8±4.7
Etiology of fever, n (%)	
Fever of unknown origin	279 (49.6)
Microbiologically documented infection	192 (34.1)
Clinically documented infection	92 (16.3)
Type of pathogen, (n, %)	
Bacteria	154 (27.3)
Virus	32 (5.7)
Fungus	27 (4.8)
Duration of neutropenia (days) (Median, range)	8 (1,49)
Median (range) interval from last chemotherapy (days)	10 (0-148)
Previous history of febrile neutropenia (n, %)	337 (59)
Antifungal therapy in the past 6 months (n, %)	123 (21.8)
G-CSF use (n, %)	164 (29.1)
Steroid in the past 14 days (n, %)	226 (40.1)
Central venous catheter (n, %)	226 (40.1)
High risk in CPG (n, %)	426 (75.7)
Follow CPG (n, %)	373 (66)
Mortality within 2 weeks of febrile episode, (n, %)	21 (3.7)

G-CSF,Granulocyte colony stimulating factor

**Table 3:** Characteristics of Severe Adverse Events (n = 115)

Severe adverse events	Number (%)
Oxygen therapy	110 (95.6)
Mechanical ventilation	37 (32.1)
Shock	100 (86.9)
Admission to intensive care unit	78 (67.8)
Renal dysfunction	24 (20.8)
Liver dysfunction	8 (6.9)

**Table 4:** Comparison Risk Factors Associated with Severe Adverse Events

Variables	Severe adverse events (N= 115)	Without severe adverse events (N= 448)	P-value
Type of malignancy, (n, %)			
Acute lymphoblastic leukemia, High risk	33 (28.7)	119 (26.6)	0.639
Acute lymphoblastic leukemia, Standard risk	8 (7)	65 (14.5)	0.03
Acute myeloid leukemia	40 (34.8)	100 (22.3)	0.008
High risk group, (n, %)	105 (91.0)	321 (71.0)	<0.001
Chemotherapy-induction for leukemia, (n, %)	22 (40)	53 (25)	0.042
Treatment not according to CPG, (n, %)	54 (47)	134 (29.9)	0.01
Previous history of febrile neutropenia, (n, %)	63 (49.4)	274 (61.2)	0.492
G-CSF use, (n, %)	35 (30.4)	129 (28.8)	0.731
Steroids use, (n, %)	53 (46.1)	173 (38.6)	0.166
Central venous catheter insertion, (n, %)	83 (72.2)	143 (31.9)	<0.001
Antifungal use in past 6 months, (n, %)	29 (25.2)	94 (21.0)	0.376
Type of pathogen, (n, %)			
Bacteria	43 (37.3)	111 (24.7)	0.009
Virus	11 (9.5)	21 (4.7)	0.067
Fungus	17 (14.7)	10 (2.2)	<0.001

G-CSF, Granulocyte colony stimulating factor; CPG, clinical practice guideline; Mann-Whitney Test and Fisher's exact test

**Table 5:** Univariate and Multivariate Analysis of Factors Associated with Severe Adverse Events of Febrile Neutropenia

	Univariate			Multivariate		
Variables	Odds ratio	95% CI	P-value	Adjusted Odds ratio	95% CI	P-value
Type of malignancy						
ALL, High risk	1.11	(0.68 to 1.79)	0.646	0.78	(0.41 to 1.48)	0.448
ALL, Standard risk	0.44	(0.18 to 0.96)	0.032	0.47	(0.19 to 1.2)	0.114
AML	1.86	(1.16 to 2.95)	0.006	0.92	(0.49 to 1.74)	0.809
High risk group	4.15	(2.08 to 9.19)	<0.001	3.35	(1.56 to 7.17)	0.002
Treatment not according to CPG	2.07	(1.33 to 3.22)	0.001	2.47	(1.51 to 4.03)	<0.001
Chemotherapy – Induction for leukemia	1.79	(1.13 to 2.82)	0.008	1.02	(0.59 to 1.75)	0.945
Central venous catheter use	5.53	(3.44 to 8.99)	<0.001	4.28	(2.51 to 7.29)	<0.001
Type of pathogen						
Bacteria	1.86	(1.16 to 2.94)	0.006	1.66	(0.98 to 2.79)	0.058
Virus	2.04	(0.82 to 4.72)	0.072	2.72	(1.05 to 7.06)	0.04
Fungus	7.88	(3.16 to 20.76)	<0.001	6.51	(2.29 to 18.56)	<0.001
Lower respiratory tract infection	5.34	(2.58 to 11.04)	<0.001	2.52	(1.09 to 5.82)	0.031

ALL, Acute lymphoblastic leukemia; AML, Acute lymphoblastic leukemia

## Discussion

Our study demonstrated that one-fifth of febrile neutropenia developed severe adverse events. Factors associated with severe adverse events of febrile neutropenia were acute myeloid leukemia, lower respiratory tract infection, fungal infection, central venous catheter insertion, and treatment not according to CPG. The mortality rate was approximately 3% and all of the patients with mortality were in CPG defined high-risk group.

In our study, hematologic malignancy is the most common underlying disease of patients with FN that accounting for two third of the patients. Our time prior to admission was 1 day that shorter previous studies (Vathana et al., 2017; Sanpakit et al., 2005). Median ANC is <100 mm3 which is similar (Vathana et al., 2017; Sanpakit et al., 2005; Allaway et al., 2019).

It has been reported that risk factors for severe infection in children with cancer or HCT recipients include (Freifeld et al., 2011): chemotherapy intensity, central venous catheter insertion (Allaway et al., 2019) breakdown of skin and mucosal barriers (e.g. mucositis). In our univariable analysis study found that AML, induction chemotherapy, lower respiratory tract infection, fungal infection, central catheter use, and treatment not according to CPG are predictive factors associated with severe adverse events. Compared with the previous study, hematologic malignancy associated with poor outcomes in febrile neutropenia (Klastersky, 2004) but our study found only AML associated with severe adverse events and ALL standard risk seems to be a protective factor. Induction chemotherapy in leukemia associated with severe adverse, while the other found chemotherapy more invasive than ALL maintenance associated with adverse events (Ammann et al., 2010). The previous study found the same result that central venous catheter insertion associated with severe adverse events (Wicki et al., 2008) and some study found that presence of significant focus of infection associated with poor outcomes (Prasad et al., 2014). However, we found only lower respiratory tract infection that significantly associated with severe adverse events. Fungal infection associated with poor outcomes and survival in our study and previous study (Kobayashi et al., 2018).

Ninety percent of patients with severe adverse events were in a high-risk group defined by CPG. Also, all of the patients with mortality were in this risk group in CPG. Therefore, clinical factors using the define risk group in our institutional CPG is validated and likely to predict for an outcome in this group of patients.

The limitations of our study are a retrospective study, and some patient data may have been missing or incomplete. Our center is Thailand’s tertiary referral university-based hospital, in which most of the patients were complicated and had various medical conditions. It is possible that our findings may not be generalizable to patients with the same condition in settings other than a university-based hospital.

In conclusion, Fungal and viral infection, central venous catheter insertion, lower respiratory tract infection, CPG defined high risk and treatment not according to CPG are significantly increased risk for severe adverse events in these patients. Our current institutional CPG for FN in children was applicable and improved clinical outcome for this group of patients.
